# The Potential of Sugarcane Waste-Derived Cellulose Fibres as Haemostatic Agents

**DOI:** 10.3390/polym16121654

**Published:** 2024-06-11

**Authors:** Siobhan Malone, Ramanathan Yegappan, Amanda W. Kijas, Anna Gemmell, Alan E. Rowan, Divya Rajah, Minjun Kim, Jan Lauko, Nasim Amiralian

**Affiliations:** Australian Institute for Bioengineering and Nanotechnology, Corner College and Cooper Roads, The University of Queensland, Brisbane, QLD 4072, Australia; s.malone@uq.net.au (S.M.); r.yegappan@uq.edu.au (R.Y.); a.gemmell@uq.edu.au (A.G.); alan.rowan@uq.edu.au (A.E.R.); d.rajah@uq.edu.au (D.R.); minjun.kim@uq.edu.au (M.K.); j.lauko@uq.edu.au (J.L.)

**Keywords:** nanocellulose, haemostatic agent, haemorrhage control, blood clotting, agricultural waste

## Abstract

Haemorrhage control during surgery and following traumatic injury remains a critical, life-saving challenge. Cellulose products are already employed in commercially available haemostatic dressings. This work explores sourcing cellulose from sugarcane trash pulp to produce micro- and nanosized fibres with hydroxyl, carboxylic acid, and trimethylamine functional groups, resulting in either positive or negative surface charges. This paper assesses the influence of these fibres on multiple blood clotting parameters in both dispersed solutions and dry gauze applications. In vitro blood clotting studies demonstrated the significant haemostatic potential of cellulose fibres derived from sugarcane waste to initiate clotting. Plasma absorbance assays showed that the 0.25 mg/mL cellulose microfibre dispersion had the highest clotting performance. It was observed that no single property of surface charge, functionality, or fibre morphology exclusively controlled the clotting initiation measured. Instead, a combination of these factors affected clot formation, with negatively charged cellulose microfibres comprising hydroxyl surface groups providing the most promising result, accelerating the coagulation cascade mechanism by 67% compared to the endogenous activity. This difference in clot initiation shows the potential for the non-wood agricultural waste source of cellulose in haemostatic wound healing applications, contributing to the broader understanding of cellulose-based materials’ versatility and their applications in biomedicine.

## 1. Introduction

**Figure 1:**
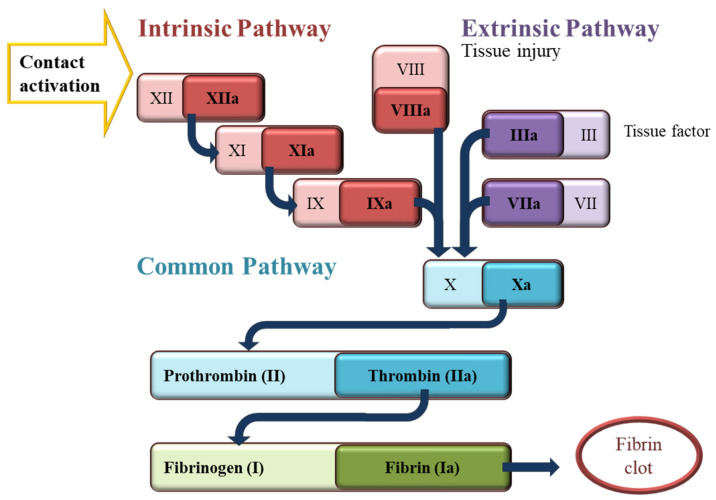
A schematic overview of the coagulation cascade. The coagulation process can be triggered through either intrinsic (red box) or extrinsic pathways (purple box). Contact with applied haemostatic agents activates the intrinsic pathway. Both pathways lead to the activation of a series of coagulation factors ultimately leading to the activation of factor X, which serves as the common pathway (green box). Upon activation, factor X triggers the conversion of prothrombin into thrombin, initiating the conversion of fibrinogen into fibrin and forming a blood clot.

Haemorrhage control following traumatic injury is a crucial concern for preventing death from blood loss in both civilian and military settings. Accounting for up to 40% of civilian mortalities and 90% of military mortalities, these deaths most often occur prior to receiving hospital care [1]. Early intervention, including the application of haemostatic gauzes and dressings, has the potential to reduce bleeding and save lives by promoting fast and successful blood clotting around the injury site. The initial stage of wound healing, haemostasis, is where haemorrhage can occur, making it a crucial target for preventing traumatic injuries from advancing to disability or death. This is the point where the signalling of the coagulation cascade is initiated to kickstart the process of blood clot formation [2] and involves numerous interacting factors and cells that can either activate or inhibit this process, including haemostatic agents (Figure 1). Activating the intrinsic pathway of the cascade through contact with certain types of artificial materials such as glass and polymers is the way haemostatic gauzes can aid in coagulation [3]. These pathways are important when considering how to activate blood and plasma clotting when a wound is created.

Some commercially available products like QuikClot^®^ Combat Gauze and XSTAT^®^ containing haemostatic agents are already on the market [4]; however, these materials come with certain limitations. QuikClot^®^ Combat Gauze, made of a polyester/rayon (regenerated cellulose) blend impregnated with kaolin as a haemostatic agent, is reported to activate factor XII [5] but lacks biodegradability, requiring its extraction from the wound [6], and there have been reports of kaolin particles entering the bloodstream and leading to distant thrombotic issues [7,8,9,10,11]. XSTAT^®^ is an injectable wound treatment comprised of compressed chitosan-coated cellulose that rapidly expands to a sponge to seal open wounds [12]. Chitosan was primarily included in the design as a nonspecific tissue glue, and now further interrogation is uncovering that it may also function as a coagulating agent by reducing antithrombin levels in the bloodstream, thereby affecting factor Xa in the intrinsic pathway [13]. Other commercially available products include, but are not limited to, Celox, WoundStat, X-Sponge, Hemcon, Chitoflex, and Bloodstop [4]. Among all of the commercially available haemostatic agents, QuikClot^®^ is recommended for paramedics and first responders [5]. However, none of these products can effectively clot a wound within a sufficiently rapid timeframe while adequately meeting all the requirements of a haemostatic agent, as highlighted by the current mortality associated with bleeding after major trauma [1]. Consequently, there continues to be a strong need for haemostatic agents that exhibit the following properties: excellent haemostatic abilities in very short timespans, low adherence to the wound and skin, acting as an effective barrier against bacteria, low cost, lightweight, biodegradable, durable, safe to use on various types of wounds, does not induce an allergic reaction in the user, and must be able to be upscaled for industrial production [14]. This study investigates the application of cellulose nano- and microfibres as new potential haemostatic agents to prevent haemorrhage and its associated deaths in the first stage of care.

Cellulose fibres in micro- and nanosizes are the main building blocks of plants, considered one of the most abundant and sustainable materials with excellent mechanical properties and tuneable morphologies. These high-performance and renewable materials have demonstrated promising potential for wound healing applications due to them being biocompatible, biodegradable, non-toxic, able to maintain surface moisture, able to absorb blood, and able to be easily removed from a wound site without causing skin trauma [15]. Based on the extraction method and resultant morphology, cellulose fibres are classified into cellulose nanocrystals (CNCs) and cellulose micro- and nanofibres (CMFs and CNFs, respectively). The CNC has a needle-like morphology with dimensions of 4–20 nm in width and 100–500 nm in length, a highly crystalline structure, and a high mechanical stiffness [16]. In contrast, CMF and CNF have both amorphous and crystalline regions in their structures, with long and flexible chains up to several micrometres in length. CMFs with a 10–100 nm width and a 0.5–10 µm length contain bundles of CNFs with diameters ranging around 4–20 nm [17,18], and some other plant structural units including hemicellulose and lignin. The high aspect ratio of cellulose micro- and nanofibres facilitates the formation of hydrogels and aerogels with a tuneable porosity, extremely low density, and high surface area [19]. Due to the available hydroxyl groups on the surface of these fibres, different surface treatments can be applied to introduce desirable functionalities required for medical applications, such as antimicrobial properties or drug delivery capabilities [14].

Recently, several research papers have explored the use of cellulose fibres, specifically nano- and micro-celluloses, as haemostatic agents [6,19,20,21,22]. An injectable highly porous haemostatic sponge was developed from the skin secretion of *Andrias davidianus* (SSAD) in combination with cellulose nanocrystals and cellulose nanofibres. The SSAD served as the bioactive component, while the CNF acted as the backbone, and the CNC assisted in achieving a uniform dispersion of the SSAD in water. The developed sponge exhibited several remarkable features, including rapid blood absorption within 3 s, shape-memory properties, high elasticity, a high blood adsorption ratio, antibacterial properties, and good biocompatibility. Additionally, the sponge demonstrated sufficient mechanical strength and high resilience after absorbing liquid.

Nanocomposites of different types of cellulose fibres with biopolymers such as chitosan, casein, and gelatine also demonstrated promise in decreasing the blood clotting time, where cellulose fibres’ network structure allows for a faster water absorption rate and high deformation recovery. The hydrophilic sponges of the chitosan and regenerated cellulose nanocomposite with a high porosity, high water absorption capacity, and rapid shape recovery were demonstrated to inhibit *Escherichia coli*, Staphylococcus aureus, and Pseudomonas aeruginosa, and achieved rapid haemostasis within 105 s [23]. The dynamic whole-blood clotting time test revealed that this composite sponge exhibited superior coagulation ability compared with traditional gauze and gelatine sponges. It also reported that the TEMPO-oxidised nanofibres (TCNFs) enhanced casein blood clotting by providing nucleation sites for blood clotting ions to bind to, which is essential for haemostasis. Moreover, the carboxylic group functionality of cellulose fibres accelerated blood clotting, promoted the adhesion of red blood cells and platelets, and stimulated the generation of thrombin, a vital component in the coagulation cascade [24]. The nanocomposite aerogel consisting of TCNFs, gelatine, and thrombin also demonstrated enhanced blood coagulation, as well as the aggregation of platelets and red blood cells [21]. This haemostatic agent exhibited rapid absorbability and a clotting time of 1.37 ± 0.15 min in rat liver punch biopsy models, leading to reduced blood loss. Mohamed et al. studied the haemostatic activity of non-oxidised CNFs prepared by high-energy ball milling compared with oxidised regenerated cellulose (ORC). It was found that the CNF produced by ball milling with an optimised aspect ratio and surface area strongly affected the promotion of haemostasis [25]. Nanofibres with a higher specific surface area and negative charge enhanced plasma coagulation by forming a mesh structure that entrapped platelets in between the fibrils, simulating their activation and the generation of thrombin through coagulation factors IX, XI, and XII (Figure 1). The optimisation of these properties outperformed the commercial ORC product in terms of clotting time [6,25]. They also reported that the CNF in the dry sponge form was more effective in reducing the clotting time, as demonstrated by in vitro thromboelastography [25]. While the majority of work reported the use of nanocellulose derived from wood as a haemostatic agent, in this work, we investigate the utilisation of nanocellulose derived from sugarcane waste. Non-wood cellulose resources are gaining considerable attention as promising alternatives to wood cellulose. Their versatility lies in their cost-effective sourcing as by-products or waste materials from various agro-based industries, encompassing crop farming, food processing, and horticulture. Sugarcane waste, including trash and bagasse, is an agricultural residue with abundant global availability. This abundance stems from the annual production of approximately 1.6 billion tons of sugarcane crops worldwide, yielding an estimated 279 million metric tons of waste annually. This waste can be converted to cellulose fibres for commercial products by pulping and bleaching processes at an industrial scale under more mild and environmentally friendly conditions compared with wood cellulose fibre production due to their lower lignin and higher hemicellulose contents, as well as a looser plant structure.

To explore the application of cellulose materials prepared from sugarcane trash as haemostatic agents, a series of different cellulose fibres through various chemical and mechanical treatments were first prepared to create both positively and negatively charged fibres of different sizes. The oxidation of cellulose through treatment with 2,2,6,6-tetramethylpiperidine-1-oxyl radical (TEMPO) has been well established as a facile approach to introduce a negative surface charge via the oxidation of the C6 hydroxyl groups of cellulose to anionic carboxylate groups (-COO^−^) [26]. Positive surface charges have also been easily and economically achieved through the treatment of cellulose pulp with 3-chloro-2-hydroxypropyltrimethylammonium chloride (CHPTAC), which acts by replacing any of the available hydroxyl groups of the cellulose unit with a cationic quaternary ammonium group via a multistep reaction in an alkaline medium, sodium hydroxide [27]. Utilising these two approaches in addition to mechanical fibre defibrillation through high-pressure homogenisation presents a straightforward approach for the preparation of micro- and nanosized cellulose fibres on a large scale.

## 2. Materials and Methods

### 2.1. Materials

Raw sugarcane trash was sourced from Sugar Cane Mulch Australia. Sodium hydroxide (NaOH, Chem-supply, Adelaide, Australia), glacial acetic acid (AcOH, Merck, Melbourne, Australia), hydrochloric acid (32%, HCl, Merck, Melbourne, Australia), sodium chlorite (80%, Technical Grade, NaClO_2_), sodium bromide (99%, NaBr), 2,2,6,6-Tetramethylpiperidine 1-oxyl (98%, TEMPO), silver nitrate (99%, AgNO_3_) and (3-Chloro-2-hydroxypropyl)trimethylammonium chloride (60 wt.% solution, CHPTAC, Sigma-Aldrich, Australia), sodium hypochlorite (12.5%, NaClO, Ajax FineChem, Brisbane, Australia), and sodium chloride (NaCl, Baxter, Merck, Melbourne, Australia) were used as received. Deionised (DI) water was used for all experiments unless otherwise indicated.

Whole-blood samples were obtained from the Australian Red Cross, Life Blood, Queensland (ethics approval #2018001922), containing citrate–phosphate–dextrose (CPD) as the anticoagulant. Platelet poor plasma (PPP) was extracted from the CPD-treated blood by centrifuging at 1500× *g* for 15 min and stored at –20 °C until use. Reagents used in the biological assays were 20× clotting buffer (20 × 10^−3^ M N-(2-hydroxyethyl)piperazine-N′-(2-ethanesulfonic acid) HEPES, pH of 7.4, 100 × 10^−3^ M NaCl) and 12.5 × 10^−3^ M CaCl_2_. MilliQ water was used for all biological assays.

### 2.2. Pulping and Chemical Treatment Methods

Sugarcane trash was washed with hot water (60 °C) to remove impurities and then dried in ambient conditions for 72 h before being ground into a fine powder using a cutting mill (SM 300, Retsch). The ground powder with a fibre size of 6-0.4 mm was then treated with NaOH (2 wt.%) at a fibre-to-solvent weight ratio of 1:10 for 2 h at 80 °C, and rinsed with hot water (60 °C) to remove soluble extractives and lignin [28]. The delignified fibres were then bleached using a 1% (*w*/*v*) acidic solution of NaClO_2_ at a fibre-to-solvent weight ratio of 30:1 for 1 h at 70 °C [28] before being washed with water (60 °C). The bleaching procedure was repeated twice. The resultant bleached pulp, which is called cellulose microfibre (CMF) in this work, was either freeze-dried to be used as a gauze or kept in the fridge for further chemical or mechanical treatments. Lignocellulose composition analysis was performed by Celignis Analytical (Limerick, Ireland) following the NREL standards, and the CMF was found to contain 67%, 22.8%, and 4.2% (*w*/*w*) of cellulose, hemicellulose, and lignin, respectively.

TEMPO oxidation of the CMF was performed by further diluting a 1 g equivalent of the CMF mass into a total of 100 mL of water containing 0.1 mmol TEMPO and 1 mmol NaBr at room temperature under continuous magnetic stirring [29]. An amount of 5 mL of NaClO solution (6–14 wt.%) at a pH of 10 (adjusted with 0.1 M HCl) was added to the suspended reaction mixture [29], which was stirred at room temperature for 2 h while a pH of 10 was maintained with the addition of 0.5 M of NaOH [30]. The resulting fibres, TEMPO-oxidised cellulose microfibres (TCMFs), were repeatedly centrifuged with deionised water at 20,000 rpm and 16 °C for 10 min until a pH of 7 was achieved, then it was stored at 4 °C or freeze-dried.

Cationised cellulose was obtained by adding 20 mL of 18.5% (*w*/*v*) of NaOH solution to 54 mL of 13.5 wt.% CMF. The dispersion was heated over 30 min to 60 °C, followed by a dropwise addition of CHPTAC (23 g). The reaction was stirred at 65 °C for 3 h, allowed to cool, then neutralised to a pH of 7 with 0.1 M of HCl and centrifuged to attain the cationised cellulose microfibre (CCMF), which was stored at 4 °C or freeze-dried.

### 2.3. Cellulose Nanofibrillation Using High-Pressure Homogeniser

All CMF dispersions of 0.7% (*w*/*v*) were processed to generate nanofibres with a Panda Plus high-pressure homogeniser (HPH) using one pass at 400 bar, one pass at 800 bar, and three passes at 1100 bar. The resultant samples were labelled as follows: cellulose nanofibres (CNFs), TEMPO-oxidised cellulose nanofibres (TCNFs), and cationised cellulose nanofibres (CCNFs).

### 2.4. Characterisation

The carboxyl group content for the TCMF was determined via conductimetric titration using a previously reported method [31]. Briefly, 0.01 M NaCl (5 mL) was added to 100 mL of 0.55 wt.% TCMF and stirred for 15 min before the pH of the suspension was adjusted to between 2.5 and 3 using 0.1 M HCl. The reaction mixture was then titrated with 0.04 M NaOH. Titrations were performed in duplicate, and the degree of oxidisation was calculated using Equation (1) [31], as follows:

(1) $$DO=M_{cellulose}\times C\left(V_{2}-V_{1}\right)/[w-36\times C\left(V_{2}-V_{1}\right)]$$

where *DO* is the degree of oxidation, *M_cellulose_* is the molar mass of cellulose monomer (162.14 g/mol), *C* is the concentration of NaOH (mol/L), *V*_1_ and *V*_2_ are the cumulative volume of NaOH added at the first and second inflexion points, *w* is the mass of cellulose in the solution (g), and 36 represents the difference in molecular weight between the anhydroglucose unit (AGU) and sodium salt of the glucuronic acid moiety [31].

Similarly, the degree of substitution of the CHPTAC-treated CMF (CCMF) was also determined via a conductometric titration using 50 mL of 0.1 wt.% CCMF and titrating with 0.01 M silver nitrate (AgNO_3_). The degree of substitution was calculated via Equation (2) [32], as follows:

(2) $$DS=C_{AgNO_{3}}\times V_{{AgNO}_{3}(eq)}/\left(w/M_{cellulose}\right)$$

where *DS* is the degree of substitution, *C_AgNO_*_3_ is the concentration of silver nitrate (mol/L), *V_AgNO_*_3_(*eq*) is the cumulative volume of silver nitrate at the equivalence point, *w* is the mass of cellulose in the solution (g), and *M_cellulose_* is the molar mass of the cellulose monomer (162.14 g/mol).

Fourier transform infrared (FTIR) spectroscopy was performed on freeze-dried cellulose samples using a Nicolet 5700 ATR-FTIR spectrometer with Diamond ATR-IR. Spectra were recorded over a wavenumber range of 525–4500 cm^−1^.

The zeta potential of the cellulose was determined via a Zetasizer (Malvern ZSU5700 Ultra). Nanofibre dispersions were diluted to 0.005 wt.% and placed in cuvettes (Cell DTS1070) before the zeta potential (mV) was determined at room temperature.

The morphology of the CNF samples was assessed with transmission electron microscopy (TEM). An amount of 15 μL of the dilute nanofibre dispersion was placed on the TEM grid and left to dry overnight. The grids were stained with uranyl acetate (UA) for 10 min in the absence of light. The remaining UA was removed, and the grids were viewed via TEM (HT770) at a voltage of 100 kV. The average fibre diameter was measured using ImageJ processing for 100 measurements.

The morphology of all samples was assessed with field-emission scanning electron microscopy (FESEM). The samples were then coated with 15 nm platinum and imaged using the JEOL 7100 operating at 5 kV.

The X-ray diffraction (XRD) analysis of samples was conducted on a Bruker D8 Advance X-ray diffractometer (Bruker, Karlsruhe, Germany) with a 0.2 mm slit. Graphite-filtered Cu-Ka radiation was generated at 30 kV and 20 mA. Samples were put into the sample holder and scanned over a range of 2θ = 5°–75° with a speed of 1°/min. The crystallinity index was measured using Equation (3) [33], as follows:

(3) $$\mathrm{CrI}\%=((I_{200}-I_{\mathrm{am}})/I_{200})\times 100$$

where I_200_ represents the intensity of the peak at 2θ = 22.4°, which is the main peak for the crystalline phase of cellulose, and the intensity of the peak at 2θ = 18.6°, I_am_; the valley between the crystalline peaks of the cellulose polymorph Iβ 1–10 (15°), 110 (16.6°), and 200 (22.4°) planes represent the amorphous domain [34,35,36].

Nitrogen adsorption and desorption isotherms were measured by using a BELSORP Max II surface area and pore size analyser at a liquid nitrogen temperature (77 K).

To evaluate the fluid absorption behaviour of the cellulose fibre, an equal mass of modified cellulose micro- and nanofibres was obtained. Briefly, whole blood was added to cellulose fibres until they reached an equilibrium weight. Dry weight (*W_d_*) and wet weight (*W_w_*) were recorded before and after the addition of the whole blood. This method was repeated with PBS (pH of 7.4). The equilibrium absorption ratio was calculated using the formulae in Equation (4) [24], as follows:

(4) $$Absorption\;ratio=\frac{W_{w}-W_{d}}{W_{d}}$$

In vitro plasma clotting assays were conducted in 96-well plates using a TECAN Plate Reader (Tecan M200 Pro Infinite). For each set, 50 μL reaction mixes were prepared containing the final concentrations of 0.25 mg/mL, 0.5 mg/mL, 0.75 mg/mL, and 1 mg/mL for each cellulose fibres’ dispersion in water, 1X clotting buffer (HEPES, NaCl), 12.5 × 10^−3^ M CaCl_2_, with MilliQ water accounting for the remaining volume. The control reaction mix contained 1× clotting buffer, 12.5 × 10^−3^ M CaCl_2_, with the remaining being MilliQ water. Tests were run with 50 μL PPP added to the reaction mix in each well, and without PPP, where 50 μL water was added to each well so that any background noise from the cellulose could be identified and removed from the results. The tests were also run with and without CaCl_2_ to determine if the cellulose had any inherent clotting abilities. Samples were performed in triplicate. Absorbance measurements were obtained at a wavelength of 600 nm, at 37 °C, every 20 s for a duration of 30 min, with the shaking of the plate for 3 s prior to each run.

To visualise the clotting ability of the freeze-dried cellulose fibres, 10 mg of each sugarcane-treated sample and 1 cm^2^ pieces of QuikClot^®^ gauze and standard gauze (Sentry Medical) were weighed in sterile test tubes. An amount of 50 μL of 0.5 M CaCl_2_ was added to each tube, including a tube without any gauze or fibre sample, so that the blood would be recalcified. An amount of 2 mL of blood was added to each tube. The tubes were placed inside a Ratek (OM11) Orbital Shaker at 37 °C and were photographed at 5, 10, 15, and 20 min intervals.

A viscoelastic haemostatic assay was performed using a thromboelastographic (TEG, Haemonetics 5000) for both the cellulose dispersions in water and freeze-dried cellulose fibre samples. TEG cups were loaded with 1x clotting buffer, 12.5 mM CaCl_2_, and 0.25 wt.% cellulose, totalling up to 100 μL, and 260 μL blood was added to start the measurements. MilliQ water was used as a control. The TEG analysis was stopped once the maximum amplitude (MA, mm) was obtained and the time taken to reach the maximum amplitude (TMA, min) was defined. The clot initiation time (R, min), kinetic time (k, min), and clot formation angle (α angle, degrees) were also recorded.

For the TEG analysis of the freeze-dried cellulose, 10 mg of sugarcane trash-treated cellulose fibre samples, QuikClot^®^ gauze, and standard gauze were weighed into a sterile tube, and 9 μL of 0.5 M CaCl_2_ was added to each TEG cup. The 600 μL of blood was kept at 37 °C before being added to each tube. The tubes were gently swirled for 1 min and 350 μL of the blood was added to the TEG cup, and the analysis was performed until the R, k, α angle, MA, and TMA values were obtained. A control, not containing any cellulose fibber or gauze, was also performed for comparison.

## 3. Results and Discussion

### 3.1. Preparation and Characterisation of Cellulose Fibres

**Figure 2:**
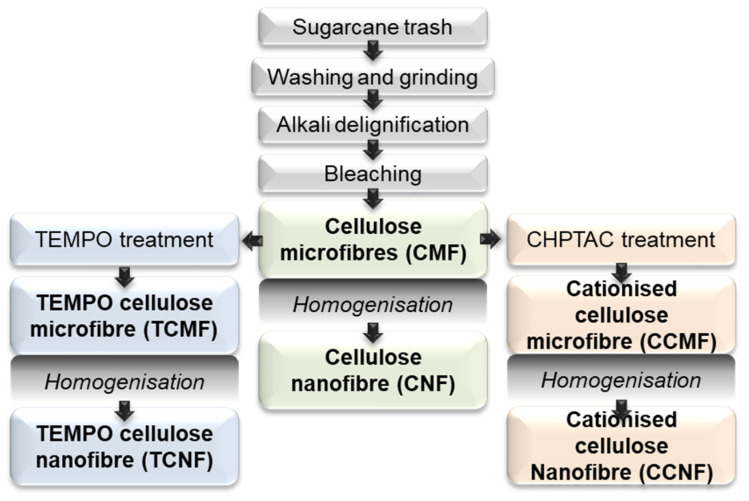
Sample preparation flowchart for the transformation of sugarcane trash into various cellulose micro- and nanofibers through chemical treatment and homogenisation.

The various cellulose materials were prepared as follows (Figure 2): cellulose microfibres (CMFs) were prepared after the delignification and bleaching of the sugarcane trash. These fibres were subjected to shear mechanical forces using a high-pressure homogeniser (HPH) to fibrillate the cellulose structure into cellulose nanofibres (CNFs). The CMFs were further reacted through either catalytic oxidation using 2,2,6,6-tetramethylpiperidinyl-1-oxyl (TEMPO) under aqueous conditions, resulting in TEMPO-oxidised cellulose microfibres (TCMFs), or reacted with CHPTAC under alkaline conditions to yield CHPTAC cellulose microfibres (CCMFs). Both the TCMFs and CCMFs were subsequently processed using HPH to generate TEMPO-oxidised cellulose nanofibres (TCNFs) and CHPTAC cellulose nanofibres (CCNFs), respectively. Following the chemical modifications using TEMPO and CHPTAC, the degree of substitution for each was determined using conductometric titrations and was found to be 0.13 mmol/g, corresponding to a 2.4% conversion of C6-OH groups on the crystalline cellulose fibril surface to carboxylate groups following the TEMPO treatment, and 0.08 mmol/g, corresponding to a 1.6% conversion of cellulose hydroxyl groups to quaternary ammonium groups following the reaction with CHPTAC. These values were assumed to be conserved following mechanical fibrillation into the TCNFs and CCNFs.

**Figure 3:**
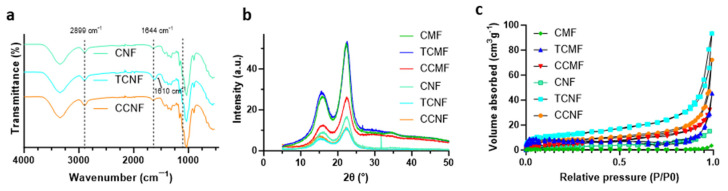
Evaluations confirming chemical modifications of the various cellulose forms: (**a**) FTIR spectra of modified cellulose nanofibres, (**b**) XRD patterns of all cellulose samples, and (**c**) BET N_2_ adsorption/desorption isotherms for all cellulose samples.

To confirm the successful chemical modification of cellulose to incorporate carboxylate groups from TEMPO oxidation and quaternary ammonium groups from the reaction with CHPTAC, the FTIR spectroscopy of the freeze-dried cellulose nanofibre samples (CNF, TCNF, and CCNF) was performed. Given the difference between the micro- and nanofibers was assumed to be purely a physical, not chemical, change, the characteristic FTIR peaks and zeta potential values of the CNF and CMF samples were expected to be the same. As shown in Figure 3a, the expected characteristic peaks of the unmodified cellulose (CNF) were observed, including peaks at 3345 cm^−1^, corresponding to the stretching vibration of the hydroxyl (-OH) groups of the cellulose backbone, the peak at 2899 cm^−1^, corresponding to the symmetrical vibrations of cellulose methylene groups (-CH), and the peak at 1644 cm^−1^, assigned to residual moisture absorbed by the cellulose. The TCNFs demonstrated a shift in peak at 1610 cm^−1^, corresponding to the newly introduced carboxylate (-COO^−^) groups. The FTIR spectra of the CHPTAC-modified sample did not show significant changes in peak intensity due to the low degree of substitution beyond the FTIR detection limit.

The XRD peaks in Figure 3b confirm the Cellulose I structure for both the micro- and nanofibres samples. Due to the mild chemical and mechanical treatments, the degree of crystallinity of all samples was found to be similar at around 70%. It was thought that the residual hemicellulose in the bleached pulp could act to protect the fibre structure during the subsequent chemical and mechanical treatments.

To confirm the intended surface charges had been imparted to the cellulose fibres following the modification reactions, zeta potential measurements of the cellulose nanofibre samples (CNF, TCNF, and CCNF) were taken. The Zetasizer can accurately determine zeta potential values for spherical nanoparticles; however, whilst the diameter of the CNF is nanometres in size, the length of the fibres can be several micrometres in length, leading to measurement inaccuracies. Despite this limitation, it was still possible to compare the zeta potential measurements amongst the samples to confirm whether the fibres contained a positive or negative charge at a neutral pH. The charge density of the CNF, TCNF, and CCNF was found to be 27 mV, −35 mV, and 30 mV, respectively.

To understand the porosity of the samples, the N_2_ adsorption–desorption isotherms were measured (Figure 3c) and used to determine the surface area of each sample. Based on the isotherms, the CMF did not exhibit noticeable adsorption patterns across the range of relative pressures, corresponding to micro-, meso-, and macropores, thus it is largely deprived of nanopores with a low specific surface area of 2.56 m^2^/g. The CNF had a slightly higher specific surface area of 8.27 m^2^/g. Upon chemical surface modification, the specific surface area of the CMF tended to increase, with the CCMF and TCMF samples determined to have specific surface areas of 15.2 and 21.4 m^2^/g, respectively. Likewise, the specific surface area of the CNF also increased to 28.1 and 41.8 m^2^/g for the CCNF and TCNF, respectively. Moreover, the N_2_ adsorption–desorption isotherms of the modified CNF (CCNF and TCNF) and CMF (CCMF and TCMF) showed significantly increased volumes of N_2_ adsorption at a high relative pressure (P/P0 < 0.9), indicating the presence of macropores.

**Figure 4:**
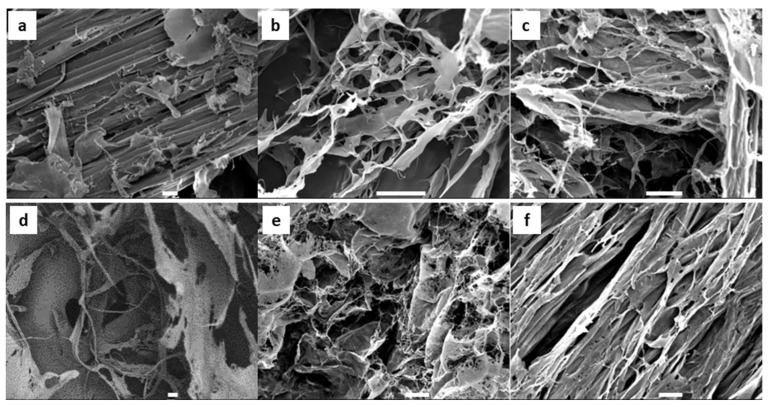
SEM images of the surface of the (**a**) CMF, (**b**) TCMF, (**c**) CCMF, (**d**) CNF, (**e**) TCNF, and (**f**) CCNF. Scale bars are 10 μm.

Cellulose fibre morphology plays a significant role in various physical properties of resultant materials, and therefore it was important to characterise its effect on the efficiency of blood clotting. The SEM image in Figure 4a shows the morphology of the CMF containing large bundles of cellulose microfibres. Upon mechanical shearing through HPH, the microfibres were expected to transition to a more heterogeneous mixture of thinner nanofibres and bundles. Due to the strong hydrogen bonding between the fibres, the nanofibers (CNFs) formed a sheet-like structure after freeze-drying (Figure 4d), and their fibrous morphology was not retained in the dry state. The SEM image of the TCMF (Figure 4b) demonstrates the random sheet-form structure with some thinner fibres. However, the TCNF (Figure 4e) formed an anisotropic porous structure with radial columnar pores after freeze-drying, indicating that possibly more ice crystals were pushed in that orientation direction due to the charge repulsion and dispersion stability of the TCNF. The directional growth of ice crystals provided aligned channels of the aerogel to form with larger pore sizes and subsequently a higher surface area, as evidenced by the BET results. The CHPTAC micro- and nanofibers (Figure 4c and Figure 4f, respectively) both had sheet-like morphologies after freeze-drying. The only noticeable differences were the thickness of the fibre sheets, which appeared to be thinner in the CCNF sample compared with the CCMF sample, and a slight difference in the porosity.

**Figure 5:**
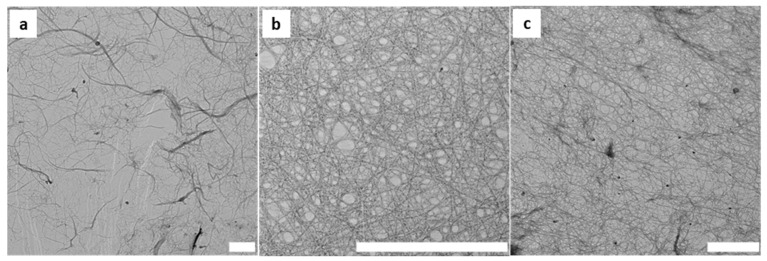
TEM images of the (**a**) CNF, (**b**) TCNF, and (**c**) CCNF. Scale bars are 1 μm.

Further investigation into the resulting fibre morphology following each chemical reaction was performed using TEM. As seen in Figure 5, the CNF contained individual fibres, and fibre bundles with a length of several microns and an average diameter of 40 ± 21 nm. Following chemical treatments, the electrostatic charges on the TEMPO and CHPTAC nanofibers were thought to promote fibre repulsion, leading to more fibrillated and individual nanofibres and a stable colloidal dispersion. This was reflected in the average fibre diameters measured for the TCNF, being 5 ± 2 nm, and the CCMF, being 15 ± 4 nm. It is of interest that, at a high aspect ratio, flexible nanofibres could be obtained from sugarcane pulp regardless of the type of pretreatment applied to the fibres.

### 3.2. Characterisation of Cellulose Fibres as Haemostatic Agents

A key mechanism of action of how materials can support the haemostatic potential is through a process of the blood factor concentration, improving the kinetics and accelerating the natural endogenous coagulation pathway. Materials that can effectively absorb wound fluids are more likely to effectively achieve the blood factor concentration. To evaluate the absorption capacity of this panel of sugarcane-derived micro- and nanocellulose products, both the simple aqueous and blood absorption capacities were compared with a predicate, QuikClot^®^ (Qc) Combat Gauze.

**Figure 6:**
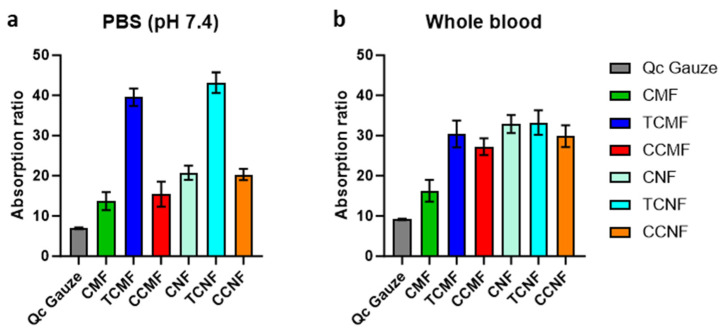
Absorption ratio of each cellulose fibre as compared with QuikClot^®^ (Qc) Combat Gauze in (**a**) PBS and (**b**) whole blood.

The fluid absorption of the cellulose fibres was evaluated through a fluid absorption assay with PBS (pH of 7.4) and whole blood, and compared with commercially available QuikClot^®^ (Qc) Combat Gauze (Figure 6). The PBS absorption assay showed that QuikClot^®^ gauze had the lowest absorption ratio of 7 ± 0.2, whilst the TCNF displayed the highest absorption ratio of 43 ± 2.6, followed by the TCMF (39.5 ± 2.2). The PBS adsorption capacity of the rest of the micro- and nanofibres was less than half of the TEMPO-treated nano- and microfibres. However, the whole-blood adsorption capacity of all of the cellulose nanofibres, as well as TEMPO- and CHPTAC-treated microfibres, produced similar results in the range of 33-29. As was seen with the PBS adsorption, the QuikClot^®^ gauze had the lowest whole-blood adsorption, likely due to the hydrophobicity of the polyester in its structure (9.14 ± 0.19), and the TCNF had the highest absorption ratio of 33.22 ± 3.04 due to the highest hydrophilicity and surface area of these nanofibres.

**Figure 7:**
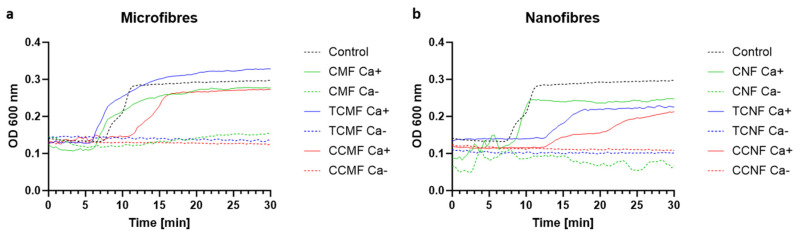
Plasma clotting assay of the control (endogenous activity) as compared with the presence of (**a**) cellulose microfibre samples of unmodified CMF, TCMF, and CCMF, all at 0.25 mg/mL with (Ca^+^) and without calcium (Ca^−^), and (**b**) cellulose nanofibre samples of unmodified CNF, TCNF, and CCNF, all at 0.25 mg/mL with (Ca^+^) and without calcium (Ca^−^).

The blood clotting performance of the materials was investigated via functional blood clotting assays using a turbidity assay, where absorbance at an optical density of 600 nm was measured over a period of 30 min to observe the plasma clotting ability of various cellulose dispersions, as shown in Figure 7. This assay was run as an initial insight into the clotting abilities of the panel of cellulose materials with an altered charge and morphology in the absence of the cell component, specifically the platelets, which play a vital role in the blood clot formation kinetics and final viscoelastic properties. Analysis of the plasma interactions focused on evaluating the activation of the coagulation pathways outlined in Figure 1 distinctly from any contribution of the platelets. In the control sample, calcium chloride was added to restore the natural endogenous coagulation pathway that required calcium for activation, and which was chelated in the CPD (citrate phosphate dextrose) blood from the Red Cross Lifeblood employed. Thus, this sample acted as the control, mimicking the physiological condition, and showed clot initiation at 7.9 ± 0.1 min through to the final clot density of 0.29 AU. As expected, the sample with no calcium control showed no clot initiation. The final clot density, however, was not the main focus of this assay due to the impact of the opaqueness and dispersibility of the cellulose solution on the absorbance within the well plates. Instead, the focus was the clot initiation time of each sample. The blood clotting performance of all sugarcane-derived cellulose micro- and nanofibres was measured in the presence and absence of calcium (Ca^+^) ions, which are required for the endogenous pathway activation, thus determining if cellulose possessed inherent clotting properties without recalcification. By plotting the difference between the wells with and without platelet poor plasma (PPP), the background caused by the opaqueness of the cellulose dispersions was removed. Concentrations of 0.25, 0.5, 0.75, and 1 mg/mL of cellulose fibre suspensions were used for this assay. Concentrations outside of this range were also analysed; however, it was found that lower concentrations did not provide any improvement in the plasma clotting time, whilst higher concentrations gave unclear results due to excess noise or inhibited the clotting through solution viscosity, and subsequently no consistent improvement was observed in the clot initiation time in response to an increase in the cellulose sample concentration, and no further analysis was undertaken. The plasma assay results for the cellulose CMF and CNF samples exhibited more noise and variation in the absorbance readings, as the dispersions were more opaque compared with the others due to the bigger fibre size. It is worth acknowledging that, as this is an absorbance-based assay, the opacity of the cellulose will affect the results through the generation of some reflectance.

Plasma clotting assays for the cellulose dispersions demonstrated that cellulose did not initiate clotting without the presence of calcium chloride and was not able to overcome any anticoagulant effects. The clot initiation time, regardless of the concentration of fibres, was seen to be 5 min for the CMF, 7.6 min for the CNF, 5 min for the TCMF, 12 min for the TCNF, 11 min for the CCMF, and 14 min for the CCNF. This shows that the CMF dispersion accelerated clotting as compared with the control representing the endogenous coagulation pathway. The TCMF and CNF dispersions also showed some acceleration. A possible explanation for this could be due to the existence of nanopores in the structure of these samples, as evidenced by the BET measurements, which act as a trap for the nanosized plasma proteins such as fibrinogen (475 A°), resulting in a more rapid fibrin formation. As shown in Figure 7, the TCMF showed better results through a faster clot initiation time compared with the TCNF and the control. The CHPTAC-modified cellulose samples, regardless of the concentration, performed poorly, with the CCNF failing to form a fully stable clot after 30 min. In addition, the cellulose microfibres in all of the samples outperformed the cellulose nanofibres, suggesting that further mechanical treatment may not be needed to achieve faster clot initiation. Of the dispersions tested, a concentration of 0.25 mg/mL was considered the best-performing sample, and therefore it was used for the whole blood-based evaluations using both a visual blood clotting assay and thromboelastographic (TEG) analyses.

**Figure 8:**
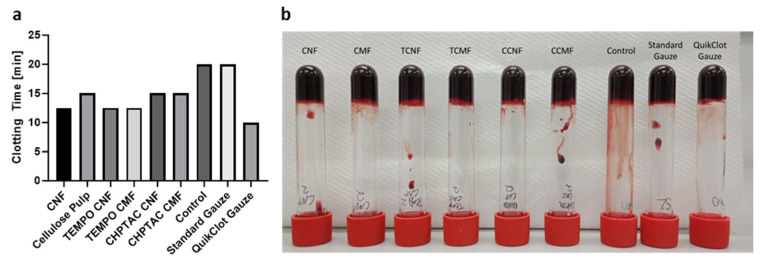
(**a**) Average final clotting time (minutes) of whole blood, performed in duplicate. Each assay contained 2 mL of whole blood, 12.5 mM CaCl_2_, and 10 mg of cellulose/gauze material. (**b**) Photograph taken 20 min after stable clot formation.

The findings from the plasma clotting assays were supported by the findings presented in the visual blood clotting assays, which displayed the time taken for complete clot formation (Figure 8). This test provided an initial insight into the nature of whole-blood clotting and the time taken for stable clot formation. It was observed that a stable clot formed between 10 and 15 min after recalcification with CaCl_2_ at 37 °C for the panel of cellulose being evaluated, which was much faster than the control, taking 20 min, but QuikClot^®^, which also contains a procoagulant (kaolin), showed the fastest time of 10 min, as shown in Figure 8a. During this assay, it was observed that the CMF and TCMF clotting tubes were the first to display signs of clotting, 10 min after recalcification, followed by the TCNF, CNF, CCMF, and CNF. Thus, it was noted that the form of cellulose, whether in a dispersed solution or freeze-dried form, influences the clotting time in whole blood.

Thromboelastographic (TEG) analysis, enabling the viscoelastic evaluation of blood clot formation to determine key clinical parameters in a precise and benchmarked approach, was performed on both cellulose dispersion and freeze-dried fibres to measure the viscoelastic properties and determine clotting kinetics and clot strength. Again, 0.25 wt.% of each cellulose was employed for these evaluations, as non-optical-based evaluations remove any of the complexity/noise observed in the plasma assays, as well as capture the key cellular contribution to the blood clots.

Several key parameters, including the clot initiation time (R time), the maximum amplitude (MA) of the clot, which is important in ensuring clot stability, the time taken to reach maximum amplitude (TMA, min), and the clot kinetic time (k, min), were measured. These parameters can inform clinicians. For example, if the MA is too low (≤4 mm), the clot would be easily disturbed and dislodged. An MA that is too high (≥75 mm) may lead to problems in patients, such as thrombus formation, blocking regular blood flow [37]. Table 1 compares the clotting kinetics of cellulose dispersions and freeze-dried fibres, respectively. In comparison to the calcium control, which triggers the body’s endogenous clotting mechanism, that initiated clotting in 14.9 min, there was a 41.6% decrease in the clot initiation time for the CMF, a 12.8% decrease for the CNF, a 32.9% decrease for the TCMF, a 21.5% decrease for the TCNF, but a 70.5% increase for the CCMF and an 8.7% increase for the CCNF. Each sample also displayed a consistent final clot density (MA, maximum amplitude) when compared with the control, and the time taken to reach the final clot density ranged between 38 and 67 min. All materials exhibited higher MA values within 16.3% of the recorded control MA, which is not a significant enough deviation in the MA to cause concern.

The TEG analysis of the freeze-dried cellulose fibre compared with the commercially available gauze demonstrated that the standard gauze performed a similar blood clotting efficiency as the control, with only a 5.8% decrease in the clotting initiation time from 13.7 min to 12.9 min. Comparatively, the QuikClot^®^ outperformed all of the freeze-dried samples, displaying a 77.4% decrease in the clot initiation time of only 3.1 min. Most of the freeze-dried cellulose fibre samples significantly outperformed the standard gauze, except for the CCNF. Specifically, a decrease in the clot initiation time of 67% for the CMF, 56.9% for the CNF, 40.9% for the TCMF, 29.9% for the TCNF, and 24.8% for the CCMF was observed.

Additionally, it has been shown throughout the plasma and blood assays that the clot initiation time and final clot density are affected by the surface functionality and morphology of the cellulose fibres. The effect of the morphology on the clot initiation time can clearly be seen by comparing the results of the cellulose networks of microfibres to cellulose nanofibres of the same chemically treated cellulose. Due to the nanopore structure of the cellulose microfibres, they entrap fibrinogen and facilitate the rapid formation of fibrin. Furthermore, when the negatively charged cellulose microfibres mix with blood, they tend to arrange themselves into a web-like network structure that has the ability to capture or trap platelets. This interaction with platelets has the potential to induce a heightened state of readiness, often referred to as “pre-activation”, which holds significant importance in the blood clotting process. In this intricate process, the generation of thrombin through the coagulation process acts as a pivotal signal, triggering the activation of platelets. The CMF derived from agricultural waste specifically contains residual amorphous hemicellulose in its structure (22.8%) (*w*/*w*), which further acts as a trap for nanosized plasma proteins, thus demonstrating the quicker blood clotting efficiency. The negatively charged cellulose fibres (CMFs, CNFs, TCMFs, and TCNFs) bond with the positively charged domains, as well as promoting the reactivity of factors IX, XI, and XII, resulting in thrombin generation and enhanced fibrin formation [38].

It is important to note that the TEG analysis of the freeze-dried cellulose and commercial gauzes has some limitations. These materials were in contact with the blood only immediately before the assay analysis. This means that they were introduced to the blood for a relatively short period, likely for the purpose of the experiment or analysis. As the freeze-dried cellulose and gauze materials were not in contact with the blood for the entire duration of the assay, the results obtained might not provide a complete or accurate picture of how these materials would affect blood clotting in a real-world scenario. Blood clotting can be influenced by various factors, and a brief exposure to the material may not capture the full extent of its impact. The assay used may be more relevant for understanding how the individual fibres interact with blood. It may not simulate the functioning of a dressing as it would be used in a medical context. Dressings are designed to come into prolonged contact with blood and wounds, and their effectiveness goes beyond just the dissociation of fibres.

## 4. Conclusions

This research has successfully highlighted the potential utility of cellulose fibres obtained from sugarcane waste as a highly effective haemostatic agent. The investigations, including plasma and blood assays, have revealed that the initiation time and final clot density could be influenced by a combination of different factors, including the morphology and surface charge of fibres. The cellulose microfibres, as demonstrated in our study, have shown great promise as a fundamental material for the development of haemostatic gauze applications. The presence of pores within the microfibre structure and their negative charge contribute to the rapid and efficient haemostatic properties. This efficacy may be attributed to their ability to concentrate coagulation factors and platelets and activate plasma coagulation processes.

These findings suggest that additional chemical or mechanical treatments aimed at altering surface functionality and morphology may not be necessary when non-wood resources are used, as the inherent properties of these cellulose fibres already lend themselves to effective haemostasis, and in combination with a procoagulant offer exciting potential. This research opens up exciting possibilities for the development of biocompatible and biodegradable haemostatic solutions derived from sustainable sources.

## Figures and Tables

**Table 1:** Measured TEG clot parameters following treatment with various cellulose fibre dispersions at 0.25 wt.%, freeze-dried cellulose fibres, and commercially available gauzes. The control represents calcium-initiated clotting (endogenous pathway).

Treatment	Clot Initiation R (min)	Maximum Amplitude MA (mm)	Time to Reach Maximum Amplitude TMA (min)
**Cellulose fibre dispersions at 0.25 wt.%**			
Control	14.9	53.9	45.5
CMF	8.7	63.1	38.6
TCMF	10	55.0	46.1
CCMF	25.4	56.2	66.5
CNF	12.8	59.0	40.0
TCNF	11.7	57.1	44.7
CCNF	16.2	56.8	46.5
**Freeze-dried cellulose fibres and gauzes**			
Control	13.7	52.8	45.9
CMF	3.8	56.6	32.3
TCMF	8.1	60.6	28.7
CCMF	10.3	56.5	40.1
CNF	5.9	54.7	33.2
TCNF	9.6	60	32.2
CCNF	13.8	52.2	44.4
Standard gauze	12.9	50.5	43.2
QuikClot^®^ gauze	3.1	62.2	26.2

## Data Availability

Data are contained within the article. The original contributions presented in the study are included in the article, and further inquiries can be directed to the corresponding author/s.
